# Region of interest optimization for radiation therapy of breast cancer

**DOI:** 10.1002/acm2.13410

**Published:** 2021-09-20

**Authors:** Tim‐Oliver Sauer, Oliver J. Ott, Godehard Lahmer, Rainer Fietkau, Christoph Bert

**Affiliations:** ^1^ Department of Radiation Oncology Universitätsklinikum Erlangen Friedrich‐Alexander‐Universität Erlangen‐Nürnberg Erlangen Germany

**Keywords:** breast cancer, radiation therapy, surface guided radiation therapy

## Abstract

**Purpose:**

The goal of this study was to investigate how the choice of the region of interest (ROI) affects the registration results of surface imaging for daily positioning of breast cancer patients.

**Methods:**

The AlignRT system (VisionRT, London) and the XVI Cone beam CT (CBCT; Elekta, Stockholm) installed on two Versa HD linacs (Elekta) were used in this study, which included 28 patients (160 fractions). In the clinical workflow, patients were prepositioned with AlignRT and then shifted in 6 degrees of freedom (DOF) according to the CBCT. A new reference capture was taken immediately afterward. Retrospectively, the surface capture resulting from prepositioning was registered to the latest reference capture. By varying the ROI used for registration, the surface‐based results were optimized in terms of minimizing the deviation to the clinically applied CBCT shifts. Two sets of ROIs were used: one obtained by applying a variable margin to the breast surface, another by combining ROIs of anatomical structures, including the sternum and contralateral breast.

**Results:**

Registration results showed significant differences from one ROI to another. Generally, the results improved with increasing ROI size, especially for rotational DOFs. ROIs, including the axilla or supraclavicular lymph drainage region, did not yield an improved registration result. On the other hand, an ROI comprising the breast surface, sternum, and a belt caudal to the breasts decreased the average magnitude of the translational and rotational deviations by 6.6% and 30.8% (*p* < 0.01), respectively, compared to the breast surface only results.

**Conclusion:**

The influence of the ROI choice on surface imaging registration results was analyzed and the surface‐based shifts were compared to clinically applied CBCT shifts. An optimal ROI for the treatment of breast cancer patients, consisting of the breast surface, sternum, and a belt, was identified.

## 1 INTRODUCTION

In recent years, surface guided radiation therapy (SGRT) has been established as an accepted alternative for daily patient positioning and monitoring.^1^ The technique generally consists of digitally reconstructing the patient's surface using a stereo camera system that detects the reflected light of a projector. This real‐time surface is registered with a previously obtained reference surface, yielding six‐dimensional shifts that correspond to the best match between both surfaces in terms of minimizing of a specific distance metric. These shifts may be used for position correction and control,^2^, ^3^, ^4^, ^5^ retrospective analysis of the patient's movement during treatment,^6^, ^7^ or monitoring of respiratory motion.^8^, ^9^, ^10^, ^11^

For systems that utilize a rigid registration algorithm like the AlignRT System (VisionRT, London), a region of interest (ROI) has to be defined by the user. The registration of the current patient surface with the reference surface is performed within this specified area only and it must, therefore, be chosen reasonably.

The breast, or respectively chest wall, is an appropriate treatment area for SGRT due to its proximity to the surface.^7^, ^12^ In spite of the wide usage of the technique,^13^ there are few and only rough recommendations, for example, by the manufacturer, for the choice of an appropriate ROI. Furthermore, there is sparse information of research on the impact of the ROI selection on the registration result available, limited namely to a paper by Alderliesten et al.^14^ The authors compared cone beam CT (CBCT) shifts to surface‐based shifts and finally recommend the usage of an ROI comprising only the ipsilateral breast and a region below it (rather than including the contralateral side as well).

In this study, the dependence of the surface‐based registration result on the choice of the ROI is being examined in detail. For this purpose, a phantom study has been carried out in a first step. The shifts obtained from the surface scanner were compared to those previously applied to a phantom by moving a 6‐degree of freedom (DOF) treatment couch to predefined positions under variation of the ROI used for registration. In a second step, surface based 6‐DOF shifts, obtained by registering surfaces of patients in treatment position to their reference surface, have been compared to the clinically applied CBCT shifts, which served as a gold standard. The choice of the ROI was optimized in the sense of minimizing the difference between surface‐based and CBCT‐based shifts.

## 2 METHODS

Treatment and phantom measurements have been carried out on two Versa HD linacs (Elekta, Stockholm), both equipped with the Hexapod treatment couch (Elekta) and the surface scanner AlignRT (Version 5.1.2; VisionRT).

### 2.1 ROIs

**FIGURE 1:**
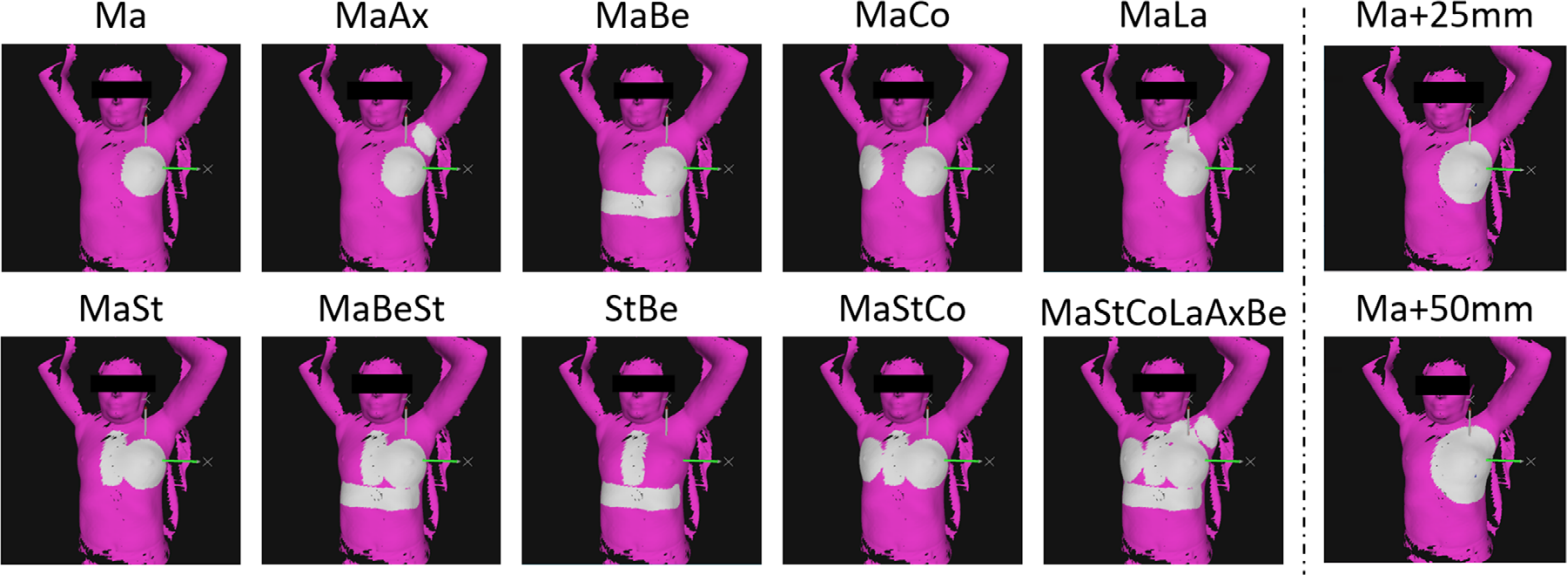
Surface ROIs. Illustration of the ROIs used in this study. To the left, ROI combinations of anatomical structures, to the right, examples of ROIs with uniform extensions are shown. Abbreviations: Ax, axilla; Be, belt underneath the breasts; Co, contralateral breast; La, lymph drainige region; Ma, ipsilateral breast; St, sternum

For the analysis of the influence of the ROI on the registration result, two sets of ROIs have been created. One set consisted of the surface of the treated breast expanded uniformly by a varying extension between 0 and 5 cm (5 mm steps). The second set, which has only been used for the patient study, consisted of different combinations made up from ROIs corresponding to the surface of various anatomical structures like the contralateral breast, sternum, or axilla (Figure 1). For matters of consistency, all ROIs were contoured by the same physicist.

### 2.2 Phantom study

**FIGURE 2:**
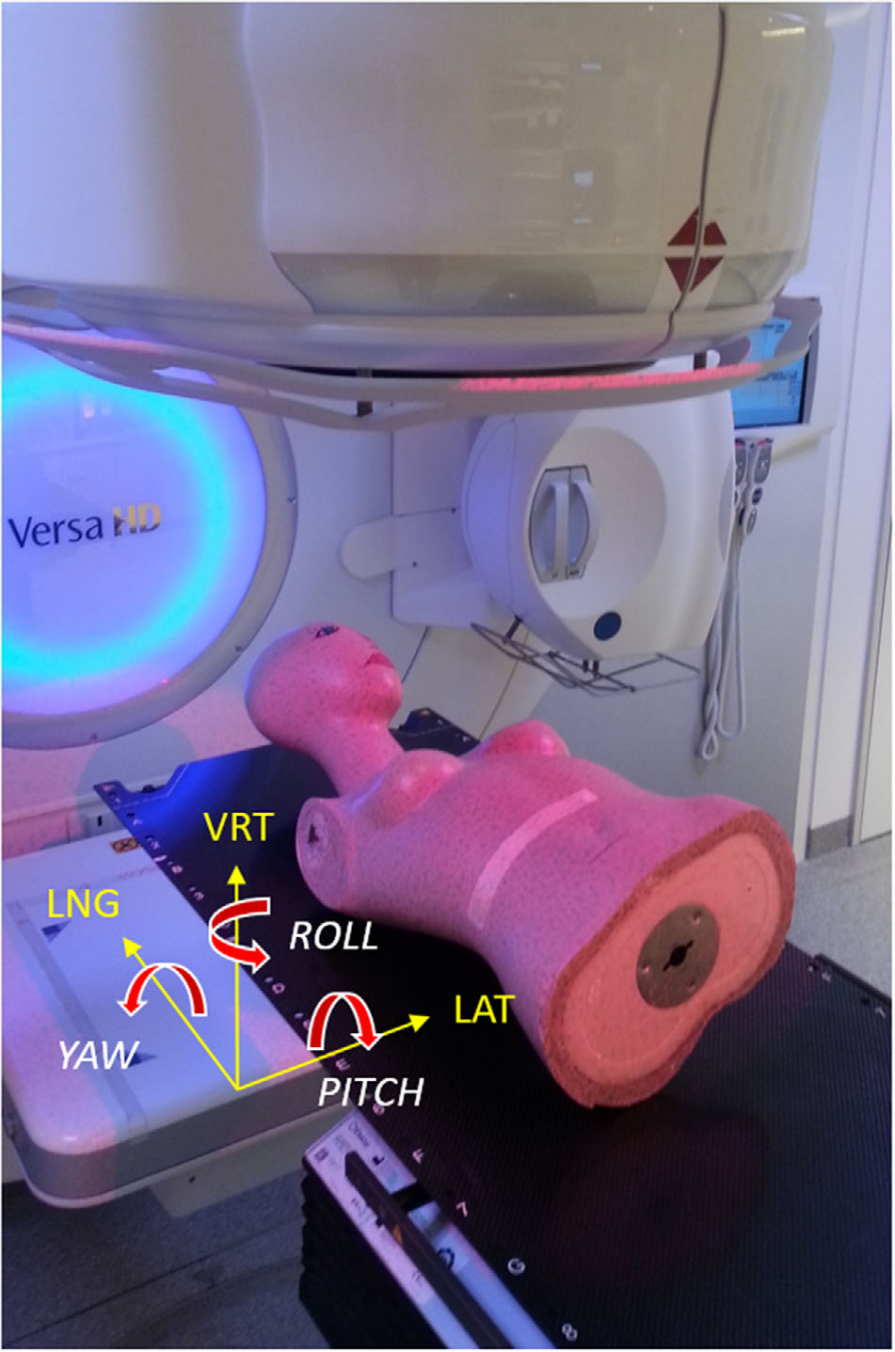
Setup. Experimental setup at the accelerator for the phantom study and coordinate system used for data analysis. The mannequin, serving as surface phantom, was placed stably in the treatment couch, which was moved to predefined positions

For the phantom study, a mannequin has been used as a surface phantom of a female patient. It was placed on the treatment couch, which is capable of moving to predefined 6‐DOF positions with submillimeter and subdegree precision, respectively^15^ (Figure 2). The movement of the phantom was analyzed with the surface scanner. Captures of the surface were taken in all positions preset by the couch. For translational DOFs, the assessed interval comprised values between –1 and +1 cm in 2 mm steps, for rotational DOFs, values between –3 and +3 degree in 0.5 degree steps. Measurements have been carried out once on both accelerators; in total, 166 captures have been acquired.

The surface captures were evaluated retrospectively using an offline program provided by VisionRT that uses the same algorithm as the online version incorporated in the AlignRT system. The offline program was chosen for the evaluation because of its capability of comparing two previously obtained surfaces retrospectively for various ROIs, with floating‐point precision output and because it is scriptable. Its registration is based on an iterative closest point algorithm^16^ with 1000 iterations for every registration (set by user). It yields 6‐DOF shifts corresponding to the optimal match of the surfaces minimizing the distance metric, which is a function of the average closest point distance of every point within the ROI. In order to assure the validity of the offline program, the determined shifts of the offline program and AlignRT were compared by registering the same surfaces (*n* = 13) with the same ROI. Both algorithms gave very similar results with mean absolute deviations of 0.2 mm and 0.06 degree for translations and rotations, respectively. The deviations are probably mainly the result of rounding differences, as the AlignRT system displays values rounded to one significant decimal place.

The offline registration was performed using the captures taken for various couch positions under variation of the ROI (breast with varying extension), using a capture taken in the initial position as reference. For every couch position, the absolute difference between the surface‐based correction shift and the predefined couch shift was calculated. Then, mean, averaged over the various measurements with different couch positions, and standard deviation of the difference were calculated for every ROI separately. Translations and rotations were examined independently from each other, meaning that only one DOF was varied at a time and only translational shifts were considered when translational DOFs were varied (similarly for rotational DOFs). This was necessary since the Hexapod treatment couch does not use the isocenter as center of rotation when shifted manually, resulting in large translational shifts when rotations are applied.

### 2.3 Patient study

In a second step, a patient study has been carried out, based on a retrospective analysis of data acquired in clinical routine. Twenty‐eight randomly chosen patients, treated in the period from 07/2019 to 12/2019, were included in the analysis (see Table 1 for patient base characteristics). Apart from fractions of the treatment of the whole target volume, only external photon boost fractions were included in the analysis as SGRT was not used for brachytherapy or electron boosts. Breast volumes were evaluated using the planning CT and automatic contouring with Syngo.via software (Siemens, Germany).

**TABLE 1:** Characteristics of the patient base used in the patient study

		Patient number
	Total	28
Treatment area	‐Left sided ‐Right sided ‐Both sided	15 12 1
	‐Breast ‐Chest wall	23 5
	‐With supraclavicular lymph nodes ‐Without lymph nodes	17 11
Treatment scheme	‐Normofractionated 28 × 1.8 Gy ‐Hypofractionated 15 × 2.67 Gy	22 6
Boost	‐Brachytherapy boost ‐Electron boost ‐Photon boost ‐None	10 4 7 7
Respiratory technique	‐Free breathing ‐DIBH	20 8
Age	‐Median ‐Range	59 34–85 years
Breast volume	‐Average ‐Standard deviation ‐Range	790 cm^3^ 493 cm^3^ 127–1828 cm^3^

During treatment, patients were prepositioned with lasers and skin tattoos. The position was corrected according to the surface‐based shifts provided by the AlignRT system. Radiation therapists (RTTs) were left to choose whether to use surface real‐time imaging or a series of single frame surface captures (called treatment captures) for position correction. In continuation, a CBCT was used for final position correction, using an automatic 6‐DOF registration to the planning CT with XVI software (Elekta) and manual correction by the RTTs, aiming for a compromise in bony anatomy and soft tissue alignment. A new surface reference capture was taken directly after the CBCT position correction at the first treatment fraction, and additionally if large deviations (larger than the clinical tolerance level of 3 mm, 3 degrees) of online shifts of the AlignRT system occurred after the CBCT‐based position correction. For patients treated in deep inspiration breath hold (DIBH), the acquisition of reference and surface captures, planning CT and CBCT was performed during inspiration; CBCTs in DIBH were paused once during acquisition. New reference captures were taken after patients had been brought to the correct inspiration level using surface guidance and their position had been corrected according to the CBCT‐based shift while still in DIBH.

A treatment capture was taken directly before the generation of a CBCT of the thorax if RTTs had chosen to use treatment captures for the initial position correction. Due to possible occlusion of the cameras and/or interference with the surface acquisition caused by the rotating gantry and imaging systems, the surface capture was not acquired during the CBCT scan. The capture was used for retrospective analysis of the ROI impact on the registration. This was done in a similar way as in the phantom study, with both ROI sets described above. Since treatment captures have not been taken every day, 160 treatment fractions were analyzed in total. The surface‐based shifts were calculated using the offline program by registering the treatment captures to the most recent clinical reference capture. The results were compared to the clinically applied CBCT shifts by calculating mean and standard deviation of the absolute difference between surface‐based and CBCT‐based shifts.

The two‐sample Student's *t*‐test for unequal variances (Welch's *t*‐test) was applied to the data in order to check for statistical significance. The test is valid due to a sufficiently large sample size (*n* = 160), even without normally distributed samples.^17^ Results were rated as not significant (*p*≥0.05), significant (*p* < 0.05), and highly significant (*p* < 0.01). All calculations and plotting were performed with Anaconda 3.1/Python 3.4.

## 3 RESULTS

### 3.1 Phantom study

**FIGURE 3:**
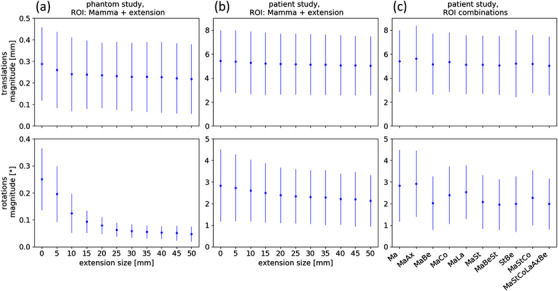
Results. Deviations (mean and standard deviation) of retrospectively calculated SGRT‐based shifts from preset couch positions (phantom study (a)) and clinically applied CBCT shifts (patient study (b) and (c)), respectively. SGRT‐based shifts were obtained for ROIs comprising the surface of the breast and a variable uniform extension ((a), (b)), or ROI combinations of different anatomical structures (c). Abbreviations: Ax, axilla; Be, belt underneath the breasts; Co, contralateral breast; La, lymph drainige region; Ma, ipsilateral breast; St, sternum

The absolute deviations of the shifts obtained from the surface capture with the offline program under variation of the ROI, averaged over the set of different couch positions (see Figure 2 for the coordinate system used), have been calculated. In general, the deviations got smaller for increasing size of the extension, especially apparent for rotations (Figure 3a). For translations, only a slight reduction of the mean deviation with increasing size of extension has been observed, stemming predominantly from the lateral DOF. Mean deviations of individual DOFs were small, almost all of them lower than 0.2 mm, with those of the longitudinal DOF being much smaller (below 0.06 mm) than the other two translational DOFs. Standard deviations were of comparable size as mean values for all DOFs and ROIs. The mean translational magnitude of the deviation decreased from 0.29 to 0.22 mm for extension sizes of 0 and 5 cm, respectively.

For rotations, a much bigger dependence of the deviations on the ROI has been observed. Mean and standard deviation decreased for all rotational DOFs with increasing extension size of the ROI, with the biggest decrease observed for the yaw DOF. This resulted in a reduction of the mean deviation of the rotational magnitude (defined in a similar way as the translational magnitude as the root mean square of the individual deviations) from 0.25° to 0.05° for extension sizes of 0 and 5 cm, respectively.

### 3.2 Patient study

For the evaluation of the patient data, the absolute deviations between surface‐based shifts and clinically applied CBCT‐based shifts have been calculated. For every ROI, the deviations have been averaged over all fractions of all patients. For a first analysis, the ROI set with varying extension has been used. The results were comparable to those obtained with the phantom, although the reduction of the mean deviation with increasing extension size was not as pronounced (Figure 3b). For rotations, the mean magnitude of the deviations decreased from 2.84° to 2.12°, whereas for translations, only a slight reduction from 5.43 to 5.04 mm has been observed. Significance was tested with Welch's *t*‐test for the samples of the magnitude of absolute difference shifts for 0 and 5 cm ROIs. The differences of the result were not significant for translations (*p* = 0.19), but highly significant for rotations (*p* = 1.31E‐4).

The analysis has been extended to the other set of ROIs consisting of combinations of different anatomical structures as defined in Figure 2. As in the above evaluations, differences were particularly obvious for rotations (Figure 3c). Compared to the ROI of the breast surface only, the ROIs that additionally include the axilla yielded larger or comparable deviations (translations and rotations). ROIs, including the lymph node region or contralateral breast, decreased rotational deviations, but increased translational deviations; those comprising the belt and/or the sternum yielded smaller deviations for both rotations and translations. For the magnitude of the translations, largest and smallest mean deviations were 5.62 mm (ROI: MaAx) and 5.02 mm (MaStCoLaAxBe), whereas for rotations, they were 2.93° (MaAx) and 1.96° (MaBeSt), respectively (see Table 2 for comparison). Welch's *t*‐test for the samples of the best‐ and worst‐performing ROI yielded a significant difference of the results for translations (*p* = 0.04) and a highly significant difference for rotations (*p* = 9.63E‐10).

**TABLE 2:** Patient study results for chosen ROIs

	Average magnitude		Relative deviation (*p*‐value) compared to Ma		Relative deviation (*p*‐value) compared to MaAx	
ROI	Translations (mm)	Rotations (°)	Translations	Rotations	Translations	Rotations
Ma	5.42	2.84	/	/	/	/
MaAx	5.62	2.93	+3.7% (0.51)	+3.2% (0.60)	/	/
MaBeSt	5.06	1.96	–6.6% (0.20)	–30.8% (1.31E‐7)	–9.9% (0.06)	–33.0% (9.63E‐10)
MaStCo LaAxBe	5.02	1.99	–7.0% (0.15)	–29.9% (2.86E‐7)	–10.7% (0.04)	–32.1% (2.48E‐9)

Note: Mean magnitude of translational and rotational deviations of surface imaging‐based shifts to clinically applied CBCT shifts for chosen ROIs, relative differences, and corresponding *p*‐value (Welch's *t*‐test).

ROI abbreviations: Ax, axilla; Be, belt; Co, contralateral breast; La, lymph drainige region; Ma, ipsilateralbreast; St, sternum.

An analysis of the results regarding the different subgroups of the patient base did not yield statistically significant differences. Stratification with respect to target volume (breast vs. thorax wall, lymph drainage region vs. breast/thorax wall only) and treatment technique (free breathing vs. DIBH) gave similar results. For all subgroups, best‐ and worst‐performing ROIs were the same. We also evaluated the dependence of the SGRT‐based shifts on the breast volume by calculating the Pearson correlation coefficient *r* of the patient‐wise averaged shift values and by performing a weighted linear regression (with the inverse of the variance as weights), using the results of the MaBeSt ROI. For rotations, we found a small positive correlation between breast volume and shift size (*r* = 0.15), with an increase of the rotational magnitude of around 0.7 ° per 1000 cm^3^ breast volume based on the linear regression. For translations, the correlation coefficient was even smaller (*r* = 0.05), with an increase of 0.05 mm per 1000 cm^3^. On the other hand, we analyzed the performance of the ROIs depending on breast volume. No breast volume threshold value could be identified below which another ROI performed consistently better than MaBeSt. Only for very large breast volumes (>1400 cm^3^), the ROI StBe had slightly better results.

## 4 DISCUSSION

### 4.1 Interpretation of results

For the interpretation of the results, it is indispensable to bear in mind the differences between the phantom and the patient study. The phantom is rigid, whereas the human tissue, especially the breast, is deformable. The results of the phantom study are, therefore, particularly interesting in order to analyze the general impact of varying the ROI, whereas the patient study gives additionally insights on the influence of deformability on the registration result of the surface scanner.

Both have in common a systematic decrease of the deviations with respect to their respective reference method (couch position and CBCT shifts, respectively) with increasing size of the ROI, particularly for rotational DOFs. This coincides with an intuitive approach to the matter: the breast is roughly a semiconvex body, which to a limited extent has spherical symmetries. If the ROI, thus the surface being registered, only consists of the breast's surface, a rotation is difficult to detect due to these symmetries. The larger the ROI gets, the more surface surrounding the breast and thus geometrically distinguishable structures are included. This is also beneficial for the detection of translational shifts, but especially effective for rotational shifts.

#### 4.1.1 Phantom study

The differences between individual DOFs in the phantom study are not interpreted as easily. It is conceivable that they originate from systematic inaccuracies either of the surface scanner, or the position of the Hexapod couch. The latter was taken as the reference in this study, although it is naturally error‐prone within a certain limit. Chung et al. reported an uncertainty for translations and rotations of around 0.1 mm and 0.01°, respectively.^15^ Based on that, the uncertainty for rotations is negligible.

The relative reduction of the mean magnitude of the deviations summed up to around 24% for translations, and 80% for rotations, comparing the ROI of the breast to that including a 5 cm extension. It has to be emphasized that the deviations in the phantom study were very small (of the order of 0.2 mm and 0.2°, respectively), but it shows the general tendency of decreasing deviations for increasing ROI size, which persists in the patient study.

#### 4.1.2 Patient study

The decrease of deviations for larger ROI sizes is less pronounced in the patient study. This is to be expected since there are many other, partly uncontrollable, factors apart from deformability that can have an impact on the registration result. The relative reduction of the mean magnitude of the deviations was around 8.9% for translations, and 25.4% for rotations, comparing the ROI of the breast to that including a 5 cm extension. There were no substantial differences between the individual DOFs, which indicates that the systematic discrepancies in the phantom study might be due to one limiting factor of the phantom study of using only one phantom and could be compensated by repeating the measurements with various phantoms. On the other hand, the absolute deviations in the phantom study were that small that they would not be recognizable in the patient study where deviations were roughly of one order of magnitude higher.

Differently to the uniform extension of the ROI, which only accounts for the ROI size, the second set of ROIs takes the location of the extension into account. The differing results underline the fact that it is important in which direction the ROI is extended. An inclusion of the axilla (“MaLa”) increased the deviation between surface‐ and CBCT‐based shifts compared to the breast surface only (“Ma”), for both translations (mean magnitude 5.62 vs. 5.42 mm) and rotations (2.93 vs. 2.84 mm). This is probably due to the deformability of this anatomical structure: the form of the breast tissue close to the axilla is very sensitive to variations in arm position^4^ and is, therefore, not suitable for positioning (see also remarks on arm position at the end of this subsection). Similarly, the ROIs comprising the contralateral breast (“MaCo”) or the region of supraclavicular lymph nodes (“MaLa”) decreased the deviations only slightly. The benefit resulting from a bigger ROI is counterbalanced here by the impact of deformability. Notably, the mentioned ROIs yielded comparably smaller rotational deviations in the direction in which they have been extended. For example, “MaAx” is an extension of the breast surface in longitudinal direction and yielded a decrease of the mean deviation for the PITCH, whereas it yields an increase in ROLL and LNG (see Figure 2 for coordinate system). This is intuitively understood, since an extension perpendicular to an axis of symmetry is beneficial for the detection of rotational deviations in this particular direction.

ROIs comprising the sternum (“MaSt”) or the belt (“MaBe”) decreased the mean deviations, especially for rotations. Here, the effect of a bigger ROI is not compensated by inaccuracies due to deformability, since these anatomical structures are comparably rigid (sternum) or deform on larger scales (belly). A combination of both (“MaBeSt”) consequently yielded the best registration results for rotations (mean magnitude 1.96°) and the second best for translations (5.06 mm). The ROI consisting of all anatomical structures used in this study (“MaStCoLaAxBe”) yielded slightly better registration results for translations (mean magnitude 5.02 mm), but slightly worse results for rotations (1.98°).

Taking into account the registration results, the mentioned difficulties due to deformability, and the fact that large ROIs lead to low framerates, the most suited ROI seems to be that consisting of the surface of the breast, including the sternum and, most importantly, the belt (“MaBeSt”). It decreased the mean deviation of the translational magnitude by 0.36 mm, thus 6.6% (*p* = 0.20, see Table 2), and that of the rotational magnitude by 0.88°, thus 30.8% (*p* = 1.31E‐7), when compared to the ROI of the breast surface only. The decrease amounted to 0.56 mm, thus 9.9% (*p* = 0.06), and 0.97°, thus 33.0% (*p* = 9.63E‐10), respectively, when compared to the results of the worst‐performing ROI (MaAx). In certain cases, a rotation of approximately one degree can already have effects on the dose distribution, especially important for large target volumes and organs at risk located far from isocenter,^18^ for instance, the spinal cord for treatments including the supraclavicular lymph drainage region. Based on the evaluation of Welch's *t*‐test, the results for rotations were considered highly significant. The results for the translations were not statistically significant and in the submillimeter range. Nevertheless, they coincide with the results obtained for rotations as to which ROIs perform best. Moreover, these values are to be interpreted as differences in the accuracy of the surface‐based registration and correspond as such to an additional offset to actually existing shifts. We, therefore, conclude that the appropriate choice of the ROI is relevant for the registration result and that the ROI “MaBeSt” is the most suited one.

Anatomical structures like the contralateral breast, axilla, or lymph drainage region should be excluded from the ROI. Nonetheless, it has been shown that the arm position has an impact on the position and form of the breast and that an SGRT‐based control of its position may be advisable.^4^ Our results indicate that (for ROI‐based systems like AlignRT) this should not be done by including it in the ROI used for positioning of the treatment area, but by using an additional ROI for the arm position (it is possible to create various independent ROIs with AlignRT). We have found that in spite of a small correlation between breast volume and shift magnitude, there is no significant influence of the breast size on performance‐based ROI selection, except that for very large volumes, excluding the breast's surface from the ROI (using StBe instead of MaBeSt) may yield slightly better results. This corresponds with the expectation that the accuracy of AlignRT's rigid registration algorithm is decreased for large and thus more deformable breasts. Due to few data (3 patients and 21 fractions), this result may not be statistically relevant though.

**FIGURE 4:**
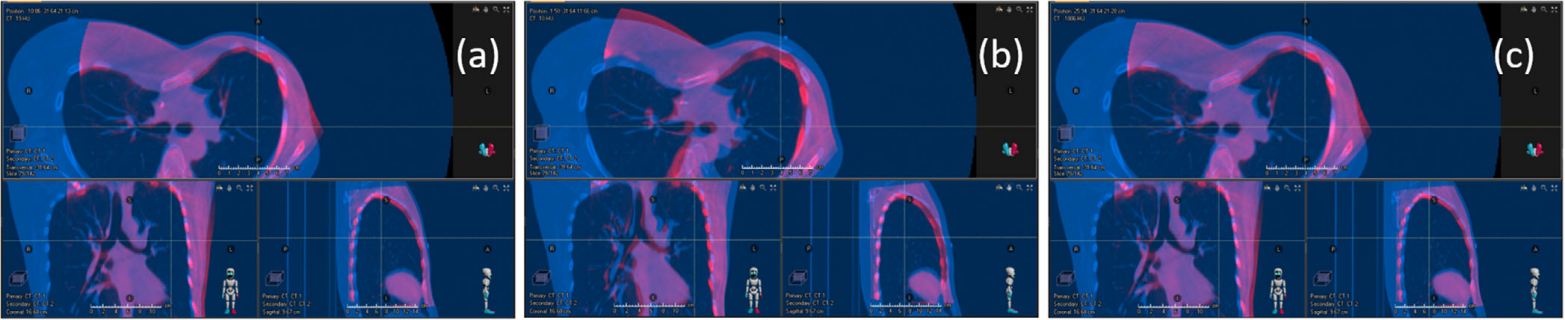
Illustration of registration results. Overlays of planning CT (blue) and daily CBCT (red), moved by clinically applied CBCT shifts (a) and surface guidance‐based shifts obtained with worst (“MaAx” (b)) and best‐performing ROI (“MaBeSt” (c)), for one exemplary fraction with large differences, performed with RayStation (RaySearch Laboratories, Stockholm)

In Figure 4, the impact of using an inappropriate ROI is illustrated. It shows overlays of the planning CT and the daily CBCT, moved by surface guidance‐based 6‐DOF shifts obtained for the best‐ and worst‐performing ROI. It is clearly visible that using an inappropriate ROI may lead to very poor registration results (Figure 4b).

### 4.2 Comparison with literature

With respect to translational shifts, the results agree qualitatively with those published by Alderliesten et al.,^14^ which to our knowledge is the only study that includes an analysis of the ROI choice on the registration. Focusing on patients treated in DIBH, the authors compared surface‐based shifts provided by the AlignRT scanner to shifts obtained from CBCT registration to bony anatomy with a bounding box (thus an ROI), including the surface of the treated breast and a region below it. For the surface registration, they used the planning CT surface as the reference and compared the result for two different ROIs, containing either only the surface of the ipsilateral breast and a region below it or both ipsi and the contralateral breasts. They found a significantly better result for translational DOF for the single‐sided ROI, which they attribute to its similarity to the ROI used for CBCT registration. We could show that their findings apply for all patients with breast cancer, independently of treatment technique and site, and additionally, that an extension of the ROI to, for example, the axilla actually impairs registration results. For rotations, they found generally poor results, and worse ones for the single‐sided ROI. Our results show that SGRT performs well for rotations too, and that the belt is the crucial extension of the ROI that leads to significantly better results. Additionally, the authors of Ref. 14 state that due to the use of a single‐camera system, large parts of the surface above the breast are not covered and therefore, roughly half of the breast was not part of the surface being registered. We have used a three‐camera system that is capable of capturing the whole breast surface and surrounding skin. The obtained mean deviations between surface‐and CBCT‐based shifts were lower than in the current study, since they did not take the mean of the *absolute* values of the deviations. The standard deviations were of the same magnitude though (around 3 mm and 2°).

### 4.3 Limitations of the study

Limitations of this study are the uncertainties of the reference methods, namely those of the position of the Hexapod treatment couch and the CBCT registration. The accuracy of the former has been determined using an IR camera system (ExacTrac, Brainlab) in Ref. 15, yielding a mean ± SD of roughly 0.1±0.1 mm and 0.01±0.01° for translations and rotations, respectively. It can thus be neglected for the interpretation of the results of the patient study (and likewise in the phantom study for rotations). The CBCT registration is user dependent and aims for alignment of bony anatomy and tissue, whereas the surface scanner naturally aligns the surface only. Moreover, the CBCT was registered to the planning CT, whereas for the surface registration, the most recent reference capture was taken, since the offline program does not accept DICOM‐extracted surface files. Treatment captures were not taken at every fraction, since the positioning method (real time or treatment captures) was chosen by RTTs. Because of this, comparably little amount of captures per patients has been taken, which is a limitation of this study.

Independently, it is not clear if the CBCT is necessarily the best method for positioning of breast cancer patients. Padilla et al. state that for near‐surface target volumes like the breast, patient positioning through alignment of bony structures using image guidance can lead to large deviations of the breast's position and that the use of surface guided positioning should be taken into consideration.^6^ Part of the uncertainties are also due to the different acquisition times of the CBCT and the surface capture, which due to occlusion problems could not be performed at the same time. A possible source of errors could be also a possibly differing order of the application of the rotations. Since the rotations are small and rotation matrices commutate for small angles, this can be neglected though.

## 5 CONCLUSION

Different ROIs have been tested with regard to their influence on the registration result of a surface scanner when compared to predefined couch positions (phantom study) and clinically applied CBCT shifts (patient study). In general, larger ROIs showed better agreement with the respective method of reference and deformable anatomical structures tended to increase deviations. Based on the results of this study, we recommend the use of an ROI comprising the surface of the breast, sternum, and a belt underneath both breasts for SGRT breast cancer patient positioning and monitoring. Other anatomical structures like the contralateral breast, axilla, or lymph drainage region should be excluded from the ROI, which is used for positioning of the treatment area. However, it might be advisable to use extra ROIs for separate positioning of, for example, the arm position. While in this study the breast has been chosen because of its SGRT‐suitability due to the near‐surface location of the target volume, the method can also be applied to other body sites.

## CONFLICT OF INTEREST

No conflict of interest.

## AUTHOR CONTRIBUTION

All authors contributed directly to the content of the paper as defined in the Authorship section of JACMP Author guidelines.

## Data Availability

The data that support the findings of this study are available on request from the corresponding author. The data are not publicly available due to privacy or ethical restrictions.
